# Investors’ exit timing of PPP projects based on escalation of commitment

**DOI:** 10.1371/journal.pone.0253394

**Published:** 2021-09-10

**Authors:** Yinglin Wang, Jingyi Chen, Jicai Liu, Chuhan Zhou

**Affiliations:** 1 Department of Management Science and Engineering, School of Transportation and Civil Engineering, Fujian Agriculture and Forestry University, Fuzhou, Fujian Province, P.R.China; 2 Fuzhou Planning and Design Institute Group Co. Ltd, Fuzhou, Fujian Province, P.R.China; 3 Department of Management Science and Engineering, School of Economics and Management, Southwest Jiaotong University, Chengdu, Sichuan Province, P.R.China; 4 Fujian Liujian Group Co., Ltd., Fuzhou, Fujian Province, P.R.China; Al Mansour University College-Baghdad-Iraq, IRAQ

## Abstract

Long project cycle and uncertainties are important characteristics of public-private partnership (PPP) projects. Since the introduction of PPP projects in China, the timing of capital withdrawal has become important. With the emergence of risk factors during the course of the project, it will face the problem of investment withdrawal by social capital financial investors. Escalation of commitment (EOC) refers to the erroneous behaviour of project decision makers who do not promptly withdraw from a project when they receive negative feedback and continue to invest resources in the project. EOC not only causes more unnecessary losses but also adversely affects decision makers. Therefore, it is crucial to clarify the impact of EOC on the choice of the exit timing of social capital. This article adopts literature survey method and quantitative analysis method: introducing the theory of maximization of income into the real option model, combining the net present value method with the binary tree option pricing model, constructing the decision-making model to analyze the exit timing of PPP social capital in the context of EOC. Then combined numerical simulation and empirical analysis to verify the effectiveness of the decision-making model, discussed the reasons why the social capital party chooses EOC, and proposes measures for controlling EOC. The higher the degree of completion of the project, the easier it is for the person in charge of the project to make inaccurate judgements about the project due to personal psychological factors, and the easier it is for EOC to occur. Therefore, after setting the minimum goal of the project, the decision maker needs to accurately evaluate the existing value of the project to avoid falling into decision-making errors.

## 1 Introduction

Public-private partnerships (PPPs) can effectively relieve the financial pressure of local governments, strengthen the circulation of market funds and reduce the risks faced by investors [1]. However, due to the long life cycle of PPP projects, risks are ubiquitous throughout the life cycle of these projects, and project risk sharing, management and control and early termination of repurchase compensation [2, 3] are complicated problems with no simple solution [4, 5].

The research shows that in many failed PPP projects, social capital parties determine that the projects will struggle to achieve the expected benefits in the early stage of the project operation phase, but they continue investing due to social pressure, risk taking [6] and the cost of input [7, 8]. Various factors cause social capital parties to choose to continue to invest in projects; this behaviour is referred to as escalation of commitment (EOC). In daily life, EOC can be seen everywhere. For example, after waiting for the bus for a long time, is it necessary to continue waiting? Do you want to continue watching a boring movie? EOC generally appears in four categories [9]:

1. Policy makers have invested considerable time and resources in a project; that is, sunk costs have been generated.
2. The project feedback received by the decision makers is negative, indicating that the project has a great possibility of failing.
3. The decision maker has the opportunity to make a second choice; that is, she can choose to continue investing in or withdraw from the project.
4. The future of the project is uncertain, and the decision makers cannot accurately estimate the final outcome of the project.

EOC is a dynamic decision-making method [10]. Decision makers are inclined to pursue opportunities [11] in projects with an uncertain future and continue to invest in these projects in order to recover current losses and obtain benefits [12]. EOC leaves decision makers unable to extricate themselves from past mistakes [13, 14]; they continue to waste resources even when there is clear evidence that the project will fail [15, 16]. Therefore, in the decision-making process of a project, it is necessary to weaken the impact of EOC and find a reasonable and effective way to help decision makers make more rational judgements.

As the main investors of PPP projects, social capital parties play a crucial role in the smooth implementation of PPP projects [17]. If private capital exits PPP projects too early, it will not only put pressure on government finance but also "tighten" the fund chain of PPP projects, forcing the projects to expand the scale of external financing. If social capital exits PPP projects too late, it will need to continuously invest resources in the failed projects, which will lead to the increase in its financial leverage and hinder the development of the enterprise itself [18]. From a macro perspective, if the social capital side fails to choose the appropriate exit time in PPP projects, the original "win-win" mode will become a "lose-lose" mode, which will have a negative impact on local employment and tax revenue [18].

Currently, in the research on the timing of withdrawal of social capital from PPP projects, it is generally assumed that decision makers are in a completely rational and ideal decision-making state, which is quite different from the actual situation. This paper introduces the factor of EOC into the choice of exit timing of PPP projects. Based on previous research results, and from the perspective of social capital, this paper analyses the EOC in PPP projects, establishes a model and identifies the optimal exit time for social capital. According to the model, the research conclusion is as follows: when the actual value of the project (including static financial value and option value) is higher than the project cost, the decision maker should choose commitment escalation; when the actual value of the project is lower than the cost of the project, the decision maker should choose to exit early rather than continue to commit to escalate. This conclusion improves the exit mechanism of PPP projects and provides decision makers with a clear and specific exit evaluation standard to avoid falling into an irrational dilemma because they cannot accurately judge the actual value of the project [19].

**Fig 1:**
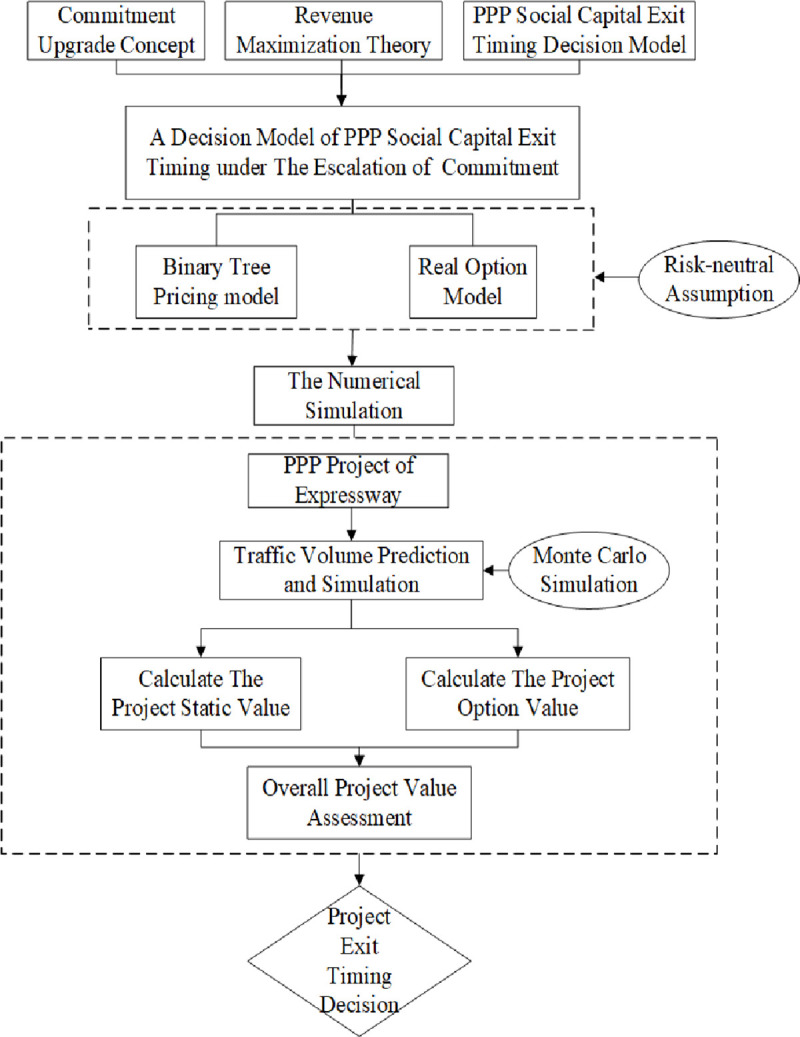
Technology roadmap. (Source: author’s work).

The technical roadmap for this paper is shown in (Fig 1):

## 2 Background

According to the World Bank PPI database, of 4,874 PPP projects in developing countries, 334 projects will be terminated before the franchise contract expires [20]. Most PPP projects have a debt ratio of more than 50%, and serious risks lead to the early termination of PPP. This early termination can be divided into two situations as follows. The first is the early termination of projects initiated by social capital to reduce income loss due to insufficient investment returns, such as low actual cash flow. The second is that to prevent the excessive profit-seeking of social capital and protect the public interest, the government initiates the process of taking over in advance when the actual cash flow is high [21]. The main factors affecting a PPP’s early termination include government decision-making mistakes, government default payments, erroneous demand forecasts, competitor projects, lack of supporting infrastructure, improper operation, insufficient financing capacity, changes in market demand, opposition from the public, policy changes, and nationalization needs [22]. Among these factors, government decision-making mistakes and government payment defaults are the most common.

For PPP projects terminated early, many scholars have proposed compensation plans and recommendations from multiple perspectives. For example, Liu et al. (2017)categorized the early termination of PPP compensation measures [21]. For projects terminated early due to social capital failures, most governments will compensate the amount of funds that an enterprise has invested in a project or compensate for the discounted value of the project’s expected future cash flow. A few governments will not compensate for national legal restrictions. If the project is stopped due to a mistake by the government, the government compensates investors according to the agreed-upon return on investment or internal rate of return or provide compensation for all losses incurred by investors. If termination is caused by force majeure, the government compensates for at least the debt of the enterprise or for the total investment amount of the enterprise. The UK Treasury requires that compensation for contractors be fair and that contractors obtain the same profit as the contract expires. This approach brings costs to taxpayers but avoids legal disputes and damage to the government’s reputation. Compensation usually includes basic priority debt termination, severance payment for project company employees, subcontracted default costs, contractor equity and the basic value of subordinated debt or compensation for open market value [23]. Xiong and Zhang (2014) introduced the two most widely used methods of early termination compensation for PPP projects [20]. First, compensation is calculated based on the accumulated cost and income before termination, which is applicable to projects based on public utilities charges and unfinished projects. Second, compensation is calculated by estimating the cash flow of the residual concession. The uncertainty is relatively large, and all long-term project risks and demand risks are borne by the contractor, which is applicable to tariff projects. Song et al. (2018) proposed a minimum guarantee and over-sharing mechanism for the early termination of PPP projects caused by government default or voluntary repurchase and divided compensation into basic and additional parts [3]. The basic salary is the minimum compensation of the enterprise, and the additional salary is a reasonable distribution exceeding the estimated profit. The aim is to ensure the smooth handover of early termination projects through pre-agreed compensation criteria both to protect the government from overcharging and to enable the private sector to receive reasonable compensation.

Reasonable early termination of projects with no profit prospects or serious losses is beneficial to contract participants. In the case of Darlington’s West Park project and the Hexham Hospital project, West Park’s contract (capital value of 16 million francs) was only 6.2 years old and was terminated at a cost of 18 million francs to achieve the expected net total savings of £14 million [24]. Hexham (capital value of 54 million francs) was terminated at a cost of 14.2 million francs, but it was estimated to save 3.5 million francs per year for the remaining 19 years of the 30-year franchise contract [25].

However, based on self-justification theory [26] and prospect theory [27] as well as government credit and public welfare, many governments prefer to commit to escalating renegotiations rather than terminating concession contracts. EOC is driven by self-defence to meet sunk costs [28, 29]. Many scholars believe that it is easier to commit to escalation in the context of decision-making dilemmas, noting that these dilemmas have the following three distinct characteristics: first, a certain amount of resources was previously invested in the project; second, the initial decision received negative feedback; and third, policymakers can choose to continue investing to recover losses or stop investing in a project and abandon it [9, 30]. Drummond (2014) examines a number of escalation of commitment cases and compares the factors that drive and reduce commitment escalation [31]. He believes that in corporate and organizational management, EOC is a ubiquitous and costly mistake. Sivanathan et al. (2008) studied the impact of the self-affirmation process on self-defence needs and commitment escalation decisions and concluded that self-affirmation psychology can be used as a tool to reduce commitment escalation [14]. Based on the theory that self-efficacy has a direct impact on the escalation of failed project commitment [32, 33], Jani (2011) proposed that risk perception moderates the impact of decision makers’ self-efficacy on the upgrading of failed IT project commitments [34].

Promised escalations are used in a wide range of applications. For example, Arbuthnott (2013) examines commitment escalation in the fossil fuel industry and infrastructure industry and advises on how policymakers can reduce commitments [35]. Liu et al.(2019) explored 18 factors affecting the commitment of investors in a PPP project and divided the influencing factors into five categories: project information and economic benefits, reward and punishment mechanisms, project uncertainty, level of participation and resources invested [36]. Through factor analysis and calculation, project information and economic benefit groups have the greatest influence on investors’ income distribution.

Currently, when studying the exit timing of social capital in PPP projects, researchers usually set decision makers in an ideal state free from external interference, and the decision of when to exit PPP projects is affected only by project income. However, the research shows that commitment upgrading has a great impact on the decision results of decision makers, so the assumption that decision makers are completely idealized will cause great deviation in research results. The existing research rarely considers the impact of commitment escalation on PPP exit timing. Early termination of a contract makes public services available at a lower cost. However, irrationally driven commitment upgrading [37] delays the best PPP termination time; this leads to the consumption of more resources at a higher cost and results in waste. Therefore, this paper holds that the decision maker’s persistence may be an irrational decision, that is, commitment escalation. Based on this, this paper builds a decision model of social capital exit timing under the condition of commitment escalation by relying on the theory of profit maximization to provide a model basis for the judgement evaluation of social capital exit timing of PPP projects, assist decision makers to make correct decisions with intuitive data, and reduce decision makers’ erroneous behaviour of blindly continuing to invest resources in the projects. Theoretical contributions of this study are mainly embodied in five aspects: (1) The research does not simply qualitatively determine the pros and cons of commitment escalation or early termination of PPP projects but employs a binary tree option pricing model with the net present value to build a new decision model to evaluate whether the decision of the commitment escalation from a quantitative point of view is rational, helping the decision maker to choose the time of exit from PPP project more objectively. (2) The research enriches the research on PPP exit timing to a certain extent. Due to the commitment to escalate the PPP project, investors cannot accurately again determine project valuation and investment cost; thus, this article introduces a real option model that not only considers the influence of the income and cost of the project but also considers the impact of future uncertainty, allowing social capital investors to evaluate the future uncertainty of decision making. (3) The study proposes that the actual value of the project includes static financial value and option value. The method of evaluating the project value based on the discounted cash flow (DCF) net present value should be improved to avoid hasty withdrawal from PPP projects due to negative feedback of the net present value (e.g., NPV<0) to make the evaluation of project value more comprehensive and accurate. (4) Based on the assumption that decision makers were rational in the past, we focus on irrational decision making, i.e., commitment escalation, which provides a new perspective for the early termination of PPP projects and is conducive to future research on more irrational decisions in PPP projects to reduce unnecessary loss of interests of the government, social capital parties and the public. (5) The research also enriches the theoretical system of commitment escalation. On the one hand, it analyses the reasons why social capital chooses commitment upgrading; on the other hand, combined with the mechanism and influencing factors of the commitment upgrade, control measures and suggestions are presented for the social capital side of PPP projects.

## 3 Research method

For the three main bodies of PPP projects, the public sector considers the possible use value of a project, while the government pays attention to the social benefits of a project. In essence, a social capital party is an economic organization with the main purpose of making profits. Safeguarding one’s own interests and achieving a profitable exit is the core of social capital investment. Driven by the theory of capital gains maximization in Western economics, the choice of the exit timing of PPP social capital is affected by the project value of the PPP project and the cost of input.

### 3.1 Model construction of exit timing selection under ideal conditions

Firm theory in Western economics focuses on the determination of product yields and prices among competing firms in the context of different levels of market competition while considering equilibrium prices. Profit *π(Q) = TR(Q)-TC(Q)*, where π is excess profit/net profit, *Q* is output, *TR* is total income, and *TC* is total cost [38].

According to the principle of profit maximization in economics, when the marginal revenue and the marginal cost are equal, that is, when *MR = MC*, the profit of a manufacturer reaches a maximum. Marginal revenue (*MR*) refers to the revenue that can be achieved by the final production or sale of a unit of product; marginal cost (*MC*) refers to the incremental cost of the final production of a unit of a product. If the marginal revenue is greater than the marginal cost, additional investment can continue to lead to profit; if the marginal revenue is less than the marginal cost, additional investment can not only increase the profit but also cause losses. Therefore, only when the marginal revenue and the marginal cost are equal will the total profit of the product reach a maximum value.

We combine the theory of vendor equilibrium with the exit timing of PPP social capital, propose a series of simplifying assumptions and construct a basic analytical framework under ideal conditions. First, we assume that social capital is the main source of increased income for PPP projects; i.e., we do not consider the contribution of other capital. Second, we assume that the benefits that the social capital party can obtain in the investment process of the PPP project can be replaced by the value of the PPP project. Third, we assume that the exit timing of social capital is not affected by the exit method and is determined only by the value of the PPP project and the cost of the input. Fourth, we assume that at any given point in time, the social capital party can make a rational and correct judgement on the value of the PPP project; that is, there is no escalation of commitment.

Based on the above assumptions, the profit maximization problem of the PPP social capital party can be expressed by the following mathematical formula:

Maxπ=TR(t)‐TC(t)=OR(t)+GR(t)−TC(t) (1)

where

*t*: investment period of social capital in PPP projects;

*TR*: total revenue function of PPP project; and

*TC*: total cost function of PPP project.

OR: operating income function of PPP project;

GR: Government compensation income function of PPP project.

Under the above conditions, the latest exit point of social capital should be when the sum of marginal operating income and marginal compensation income equals marginal cost. The mathematical expression is as follows:

MR(t)=MC(t) (2)

Studying venture capital, Cumming and MacIntosh (2001) [38] proposed that *MR* (the project marginal revenue function) has a higher value at the initial stage of investment. With the increase in investment period *t* and the gradual improvement in PPP projects, the marginal revenue will gradually decrease, which is consistent with the law of diminishing marginal revenue in the principle of maximizing returns. Similarly, *MC* (the marginal cost function of input) shows a downward trend in the growth range of the investment period because the total cost (*TC*) of PPP projects is usually divided into fixed and variable costs. Under the marginal cost method, the model considers only the variable cost of the last additional unit. As the investment period grows, the total cost of the project gradually approaches the fixed cost; that is, the slope of the marginal cost of the project approaches 0.

The intersection point of the *MR* curve and the *MC* curve is a key node in the selection of the exit timing of PPP social capital. At the initial stage of PPP social capital investment, that is, before *t*_0_, the *MR* function is higher than the *MC* function, indicating that the added value of the expected revenue of PPP project is higher than the expected cost function and the project gains. After *t*_0_, the *MR* function is below the *MC* function, and the project is in a loss state. A rational investor should withdraw from the project in time to prevent losses. That is, point *t*_0_ should be the latest exit point of PPP social capital.

**Fig 2:**
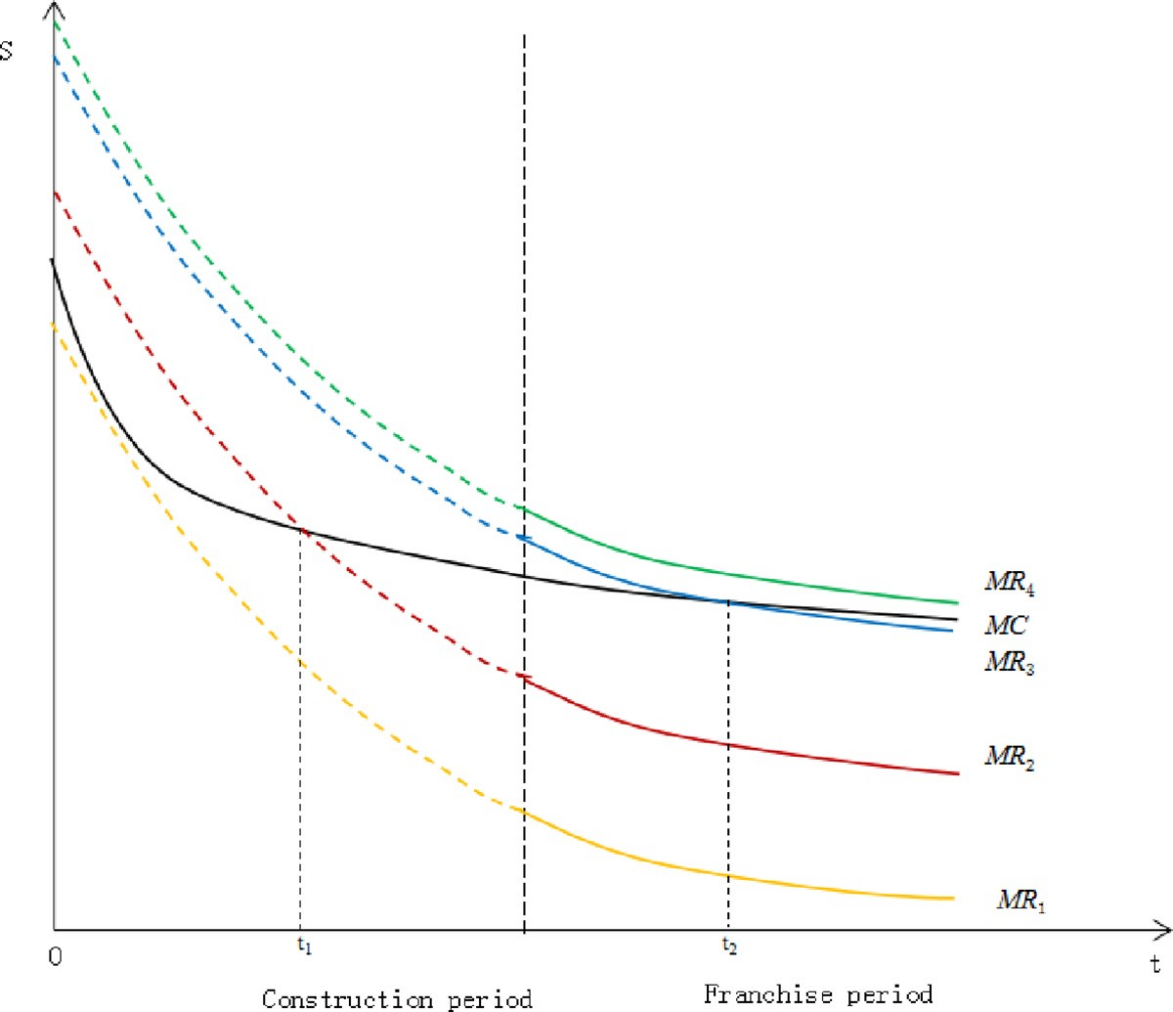
The intersection of *MC* and *MR* curves. (Source: author’s view).

If the construction period and franchise period are divided into two stages, the graph drawn in (Fig 2) can be obtained. During the construction period, there is only cost input and no revenue generation, so there is only a decreasing *MC* curve. Until the end of the construction period and the start of the franchise period, the project gets operating income and government subsidies, and the *MR* curve is generated. The intersection of *MC* and *MR* can be divided into three situations: (1) There is no intersection point between *MC* and *MR* curve, as shown by *MR*_*1*_ and *MR*_*2*_ curves. The MR curve has always been lower than the MC curve since the operation period, which means that investors should exit the project during the construction period; (2) There is no intersection between *MC* and *MR* curve. As shown by *MR*_*4*_ curve, *MR* curve has always been higher than *MC* curve since the beginning of the operation period, which means that the project is in good operation condition and social investors do not need to quit the project; (3) There is an intersection point between *MC* and *MR* curve. As shown by *MR*_*3*_ curve, *MR* curve intersects *MC* curve at some point during the franchise period, which means that social investors should exit the project at the intersection.

### 3.2 Model construction of exit timing selection under escalation of commitment conditions

In PPP projects, due to a large number of sunk costs and the existence of government guarantee, investors often choose EOC rather than exit early. Unless the loss after EOC intensifies and exceeds the psychological threshold of investors, investors will exit the project in advance to avoid greater losses. Due to the uncertainty and risks implied in the PPP projects, too much loss in this project, is unable to continue under the condition of construction or operation, investors tend to have early exit, the project is handed over to the rights of the government to take over and there is no corresponding obligation, this behavior can be regarded as put options, give up the option to perform investor’s early exit as. So it can be argued that investors have autonomy in their choice of exit timing.

We relax the fourth hypothesis of the exit timing selection model under ideal conditions and consider the impact of commitment escalation on the exit timing of social capital.

1) Escalation of commitment impacts estimation of project value

Traditional project static valuation methods often ignore the potential benefits of risk. If the value of a project is simply equivalent to the net present value (*NPV*) of the project, when the *NPV* <0, the project loses investment value and investors should immediately withdraw from the project. Under EOC theory, a social capital party believes that the value of a project includes not only the *NPV* of the project but also the uncertainty value of the project; that is, the value of the project will increase to a certain extent.

**Fig 3:**
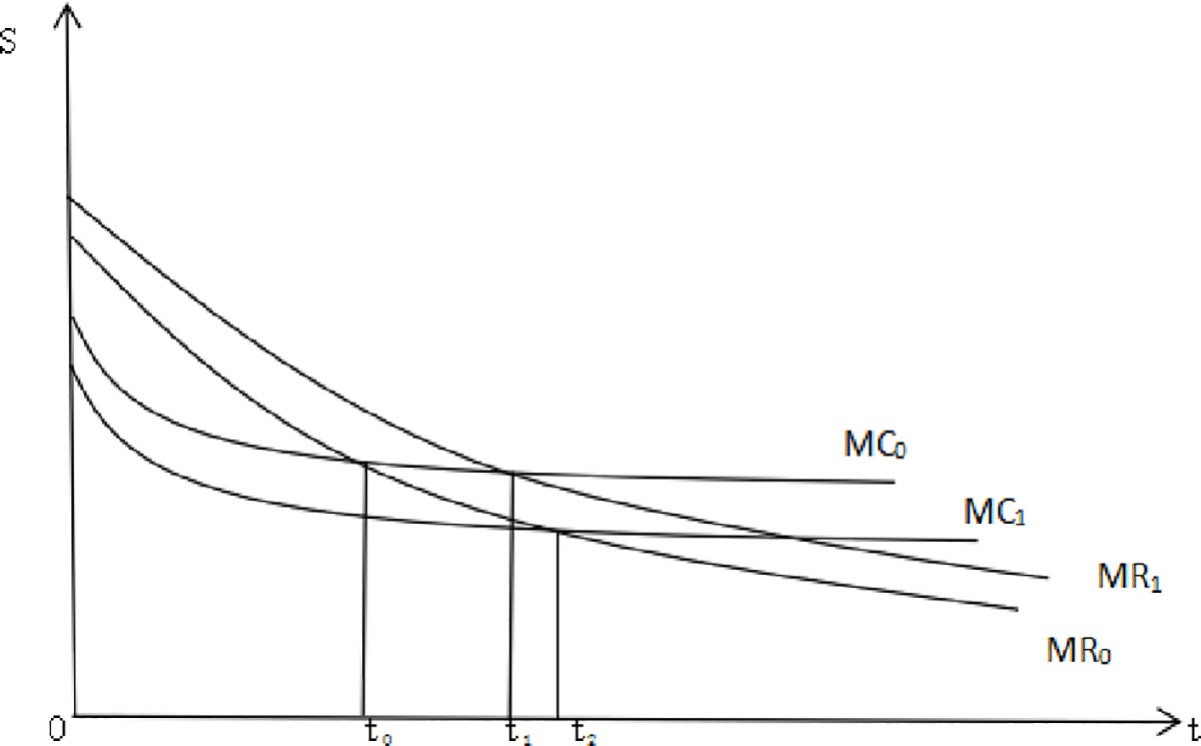
Comparison of the timing of social capital exit. (Source: author’s view).

In the principle model, the real value of the project is replaced by the marginal revenue function. It is not difficult to find that the existence of EOC improves the value of PPP projects, and the added value is brought by the uncertainty of the project after choosing to escalate commitment. Under the same conditions, the *MR*_0_ function curve should be redrawn into the *MR*_1_ curve. The investment period of PPP social capital increases; that is, the point moves backward when exiting and retreats from *t*_0_ to *t*_1_ (Fig 3).

2) Escalation of commitment impacts the cost estimate of the project

Prospect theory is the most important of the many theories explaining and analysing commitment escalation behaviour [39]. In the foreground theory, decision makers have their own criteria for evaluating the value of the project and the cost of investment. They consider not only economic factors but also social, psychological and environmental factors. The project value function curve of the foreground theory changes in an S-shape, and the slope value in the loss region is significantly higher than that in the yield region. Kahnemen and Tversky (1979) argue that when a project is in a different judgement framework, investors’ attitudes towards risk holding are also significantly different [27].

There is a marginal diminishing effect under two different frames. When the decision maker is in the frame of revenue, the decision maker’s subjective revenue is less than the objective revenue. When the decision maker falls into the loss framework, she subjectively feels that the loss is greater than the objective loss value. At this time, the decision maker tends to pursue the risk, and an external challenge is regarded as an opportunity. According to the marginal diminishing effect, the decision maker’s understanding of the value of the project input cost deviates, and the value of input cost is underestimated. In other words, on the premise that other conditions remain unchanged, the *MC*_0_ curve will move downward to *MC*_1_, making the investment period of the social capital party of the PPP project increase from *t*_0_ to *t*_2_ (Fig 3).

A comprehensive analysis of the above two situations reveals that under the effect of EOC, different analysis methods lead to different function curves being redrawn, but the result is that the investment period of the project is prolonged. Under the EOC theory, the social capital party pays more attention to the uncertainty value in the project. When the project has negative feedback, the social capital party thinks that it can still continue to invest in the project and that the project has potential value. Then, in the eyes of those who choose EOC, the actual value of PPP projects should also include the uncertain value of the projects’ future. In PPP cooperation, the government usually promises some guarantee measures, such as minimum vehicle flow guarantee and minimum income guarantee, etc. However, due to the complexity and risk of the project, some guarantee measures fail to achieve the expected effect, resulting in less than expected profit or even loss of the project. Therefore, the value of project uncertainty is closely related to the level of government guarantee. Formula (1) can be extended to obtain

Maxπ(t)=TR(t)−TC(t)+gQ(t) (3)

where *g* is the coefficient of government guarantee, Q is the uncertain value of the PPP project in the future.

Under commitment escalation, the value brought by the future uncertainty of the project occupies an important position in the actual value of a PPP project. The exit timing of the social capital party is influenced not only by project income and cost but also by the value of uncertainty. Then, in the eyes of those who choose to escalate their commitment, what is the uncertainty value of a PPP project? How can the value of uncertainty be determined? At this time, it is very important to establish an analysis model that can be used for reference by social capital parties to transform investors’ subjective feelings into objective data and provide accurate termination threshold for social capital parties, so as to reduce commitment escalation behavior.

## 4 Research steps

### 4.1 Theoretical basis

For a long time, PPP projects have typically used discounted cash flow (DCF) to evaluate their value. However, due to the lack of alignment between the assumptions made by the DCF method and the actual situation, the results are prone to large deviations [40]. This situation often leads the social capital party to underestimate the project value and to ignore the positive significance contained in project uncertainty [41].

The concept of real options is derived from financial options [42]. Real options refer to the uncertainty value hidden in a project. This value may be large or small, as determined by the characteristics of the project. The greater the uncertainty of the project, the higher the option value. To date, real options have formed a relatively complete theoretical system. According to their nature, options are divided into call options and put options. Delayed options, abandon options [43], growth options, expansion options, contracts, conversion options, chooser options [44], compound options [45], and other forms of general options are derived from the two major types of options [38]. A real option is a flexible investment decision concept, and this method can accurately evaluate the value of a project. In 1973, Black and Scholes put forward the famous B-S option pricing theory, which laid the foundation for option pricing theory [46]. Then, in 1979, Cox et al. used a relatively simple method to derive the binomial tree pricing model, also known as the binomial model [47]. The intuitive and simple nature of the binomial tree model caused it to be widely applied and extended.

Real options have advantages that traditional DCF methods cannot match. From the perspective of real options, the real value of a project also includes the option value due to the uncertainty of the future. At this point, if the static investment value of a project *NPV*<0, the social capital party does not have to exit the project immediately. Similar to real option theory, EOC is a dynamic decision-making [4] method that gives decision makers a more flexible decision-making scope and future uncertainty as opportunities for development. This paper introduces the binomial tree pricing model of real options considering that in the case of a commitment to escalate, the social capital party still invests resources in the project and does not leave the PPP project when the *NPV*<0.

### 4.2 Assumptions of the model

Assumption 1: To introduce the binary tree option pricing model, a risk-neutral hypothesis is proposed. The risk-neutral assumption is the basic assumption premise of various real option pricing models. Under the risk-neutral condition, investors do not require any risk compensation, and the expected rate of return is risk-free interest, which is a fixed value and does not change before the end of the franchise life.

Assumption 2: Within the remaining franchise years of the PPP project, the value of the project has only two possibilities for change: up and down. The probability and amplitude of each fluctuation remain the same; that is, if the probability of rising is *p*, and the probability of falling is *1-p*.

Assumption 3: When the PPP project faces a new decision point, based on the model of the binary tree option multi-period pricing, the remaining franchise period of the PPP project is equally divided into n time intervals, namely, $\Delta t=\frac{T}{n}$. This paper assumes that each time interval of the project is 1 year, Δ*t* = 1.

### 4.3 Model construction

If there is negative feedback when the PPP project reaches the t-th year, the social capital party faces two choices. The first is to adhere to the project and escalate commitment so that the project’s later income can reach the expected return. The second option is to withdraw from the PPP project in advance. Comparing the EOC theory with real option theory, this paper asserts that the actual value of a PPP project should be composed of the static financial value and the option value of the project [48].

$$V=V_{0}+gV_{1}$$ (4)

where *V*_*0*_ is the static financial value of the project, namely, NPV, and *V*_*1*_ is the project option value, namely, the uncertain value.

#### 4.3.1 Static financial value of the project

The PPP project is mainly divided into the construction phase and the franchise operation phase. Most of the early withdrawal of social capital occurs during the franchise period of the project [49]. Therefore, this paper studies how to choose the exit timing of social capital in PPP projects only during the franchise period and considers whether EOC should be chosen at this time. The formula for calculating the net present value of the project is

$$NPV=\sum_{i=0}^{n}\frac{CI_{i}-CO_{i}}{{\left(1+r\right)}^{i}}$$ (5)

where n is the total life period of PPP projects, including the construction period and franchising period;

*CI*_*i*_ is the cash inflow of the PPP project in the i-th year;

*CO*_*i*_ is the cash outflow of the PPP project in the i-th year; and

*r* is the discount rate of the project.

#### 4.3.2 Project option value

**Fig 4:**
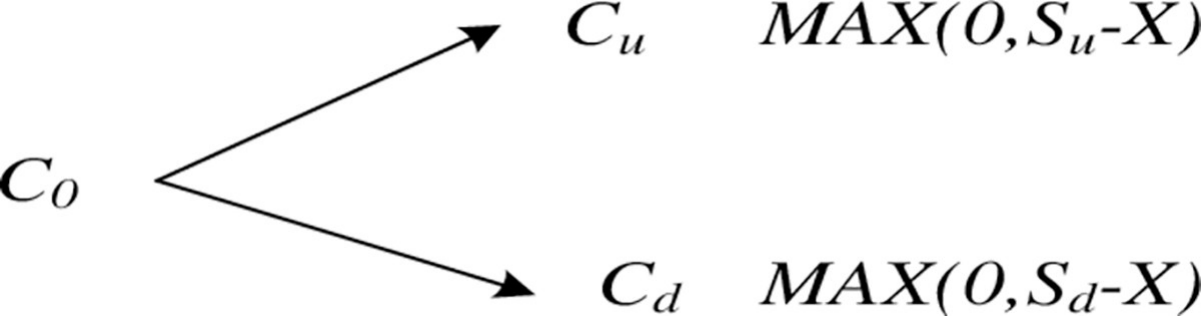
Schematic diagram of a single-stage binary tree. (Source: author’s work).

In the binary tree model, it is assumed that the initial value of the PPP project is *S*_*0*_. In the period between the option expiration, the probability that the value of underlying asset *S*_*0*_ has *p* rises to *uS*_*0*_ and the probability of *1-p* drops to *dS*_*0*_. *X* refers to the option strike price of the project (Fig 4).

**Fig 5:**
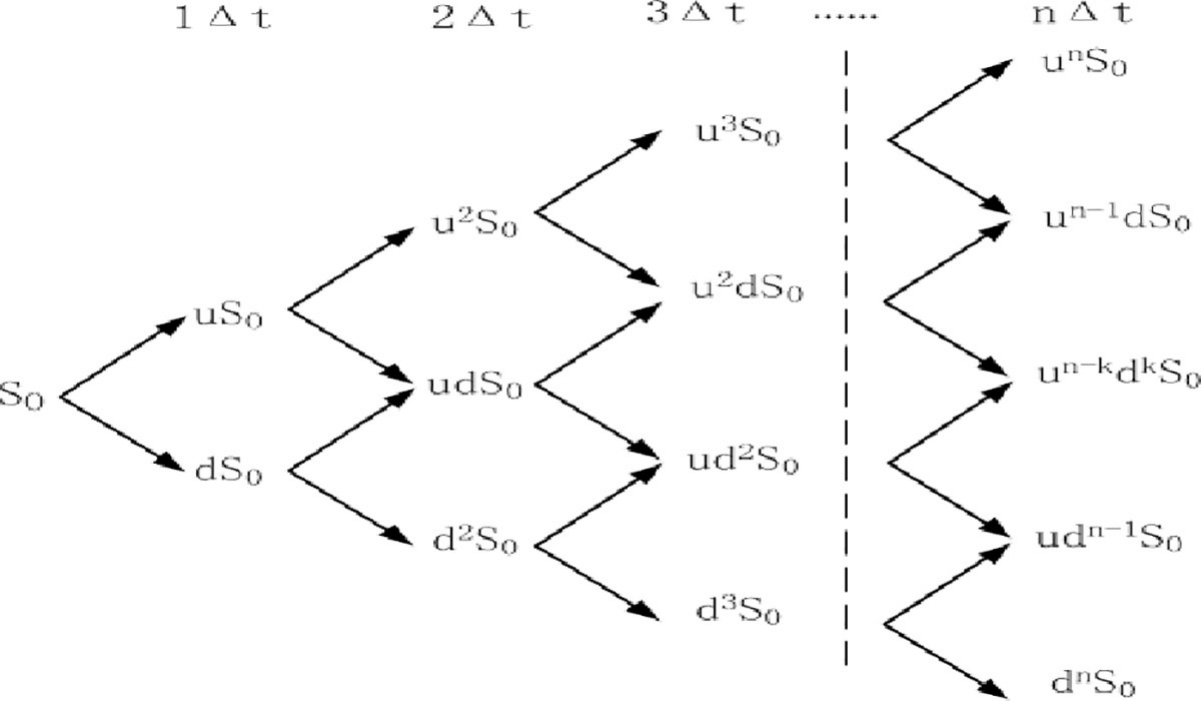
Schematic diagram of multi-stage binary tree. (Source: author’s work).

The core idea of the binary tree option pricing model is to discretize the option duration (the franchise period of PPP projects) into multiple nodes according to the process shown in (Fig 5). At any one node, the two possible values for the next time period of underlying asset *S* are *uS* and *dS*. The franchise period of PPP projects is generally 20 or 25 years, with a longer option duration. Obviously, using a single-ended binary tree model to describe the option value of a project is not accurate. Therefore, according to certain rules, the single-phase binary tree model is extended to the multi-phase model (Fig 5).

The formula for the binary tree model is as follows:

$$C=e^{-r_{f}\Delta t}[pCu+(1-p)Cd]$$ (6)

$$P=\frac{e^{r_{f}\Delta t}-d}{u-d}$$ (7)

where

*C*: option price, which is replaced by the uncertainty value in this article;

*C*_*u*_: value of the option in the upward period at the expiration date;

*C*_*d*_: value of the option in the down period at the expiration date;

*p*: risk-neutral probability;

*r*_*f*_: risk-free interest rate;

*u*: price upward multiplier, $u=e^{\sigma \sqrt{\Delta t}}(u>1)$; and

*d*: price down multiplier, *d* = 1/*u*(*d*<1).

We use this formula to reverse the value of the expiration date of the option and calculate the option value of each period of the project until the option value at the initial moment of the project is obtained. Therefore, the present value of an option is essentially the discounted value of the expected present value of future options at a risk-free rate.

### 4.4 Parameter determination of the model

According to the well-known B–S pricing formula [46], the meanings of the model parameters *S*, *r*_*f*_, *X*, and σ can be determined as follows:

1) Project initial value S

The initial value of project *S* refers to the present value of the expected return of the project, that is, the sum of the present value of all future cash flows [50], and the discounted value of the future cash flow discounted at the risk-free rate.

2) Risk-free interest rate *r*_*f*_

The risk-free rate is an important parameter for calculating the value of an option. It refers to the rate of return on capital investment in a risk-free investment project [50, 51]. For the sake of calculation simplicity, the risk-free rate usually uses the long-term government bond interest rate issued during the same period.

3) Option strike price *X*

Strike price X is expressed in the formula as the input cost of the project [50], which is equivalent to the subsequent investment cost of the project in the face of re-selection. If the decision maker chooses to exit the project, there is no secondary investment cost and no escalation of commitment. If the decision maker chooses to continue investment and escalate commitment, the investment cost at this time is equivalent to the decision maker’s commitment escalation cost.

4) Volatility σ

Volatility is the range and frequency of price changes and the volatility and uncertainty of earnings [50, 52]. The choice of volatility directly affects the price up and down multipliers of the option pricing model. Usually, the value of an option increases as volatility increases. In PPP projects, the most commonly used calculation methods are historical volatility and implied volatility. This paper uses the historical volatility method to estimate the volatility of the asset price of a PPP project. The calculation method is based on the historical asset data of a PPP project to calculate the standard deviation of the price return rate. The calculation steps are as follows:

①Collect the value *S*_*i*_ of the target item at a fixed time period (this article selects an annual time interval)
②Calculate the natural logarithm of the ratio of the value of the time period to the value of the previous time period, $u_{i}=\ln\frac{S_{i}}{S_{i-1}}$
③Calculate the standard deviation of these natural logarithms, and multiply it by the square root of the number of time periods in the number of years. The specific formula is as follows:

$$\sigma =\sqrt{\frac{\sum_{i=1}^{n}{\left(u_{i}-\bar{u}\right)}^{2}}{n-1}}$$ (8)

## 5 Numerical simulation

### 5.1 Basic data

According to the model constructed, numerical simulation is carried out to further reveal the social capital exit timing of PPP projects under the influence of commitment escalation. Although the value of each parameter and the calculation method of the constructed model have been clarified, the numerical simulation analysis method can not only offer a clearer understanding of the relationship between each parameter but also facilitate a more intuitive discussion on whether a project chooses to escalate commitment.

A province uses PPP to build a new expressway with a total length of 120 kilometres. The total investment during the construction period is 10 billion yuan. The construction period is 5 years. The annual investment ratio for years 1–5 is 15%, 25%, 30%, 15% and 15%, respectively. The franchise period agreed between the government and the social capital party is 20 years. The social capital party can obtain proceeds by collecting fees from passing vehicles during the franchise operation period. After the end of the operation period, all fixed assets must be handed over to the government without compensation. In the fifth year of the project operation, due to the over-optimistic prediction of the passenger flow of the expressway, the actual income of the social capital party during the operation period is lower than the expected income. Therefore, the social capital party needs to analyse the current situation and choose whether to escalate its commitment.

Assumption 4: The social capital party can carry out effective cost management during the franchise period of the project; that is, the total investment during the construction period of the project is directly proportional to the quality of the PPP project and inversely proportional to the annual operating cost during the franchise period, which is expressed as *M* = *kC*^−*a*^(*k*>0,*a*>0) where *M* is the annual operation and maintenance cost of the project during the franchise period; *C* is the total investment of the project during the construction period; and coefficients *k* and *a* are determined according to previous similar projects, taking 600 and 1.1, respectively. After calculation, the average annual operating cost is

$$M=600\times 100^{-1.1}=378.5744(\mathrm{Million}\;\mathrm{yuan})$$

Assumption 5: the discount rate of this example is determined using the CAPM (capital asset pricing model), and the model formula is as follows:

$$CAPM=r_{f}+\beta (r_{m}-r_{f})$$ (9)

where *r*_*f*_ is the risk-free rate of return that adopts the interest rate of the maturity of long-term Treasury bonds, which is 5%. *β* is the risk correction coefficient, which is 0.6, and (*r*_*m*_-*r*_*f*_) is the risk premium of the market, which is 6%. After calculation, the discount rate of this paper is

r=5%+0.6×6%=8.6%

Assumption 6: according to the deviation between the actual passenger flow and the expected passenger flow, the coefficient of government guarantee *g* is assumed to be 0.35.

According to the road toll standard of G15 Shenhai Expressway (formerly Funing Expressway) in Fujian Province in 2018, the traffic types and charging standards of the expressway are shown in (Table 1), without considering additional fines or concessions.

**Table 1:** Vehicle charging standard.

Model	Truck	Bus	Charge rate (yuan/car/km)
First class car	Less than 2 tons (including 2 tons)	Below 7 seats (including 7 seats)	0.6
Second class car	2 tons—5 tons (including 5 tons)	8–19 seats	1.2
Third class car	5 tons—10 tons (including 10 tons)	20–39 seats	1.8
Fourth class car	10 tons to 15 tons (including 15 tons) and 20 feet container carrier	Over 40 seats (including 40 seats)	2.1
Fifth class car	Over 15 tons and 40 feet container carrier		2.7

Source: Traffic Violation Inquiry Network http://www.chajiaotong.com/fagui1/85312.

To simplify the calculation, the traffic volume of the project is uniformly converted into the tolls for one class of vehicles according to the charging standards in the table. Each car is set to travel 120 km; that is, the toll for one car is 72 yuan.

We collect the actual data for the first 12 years of similar projects (Table 2) to predict the traffic flow during the remaining concession period.

**Table 2:** 12-year actual data for similar projects.

Year	Traffic volume *Q* (10,000 units)	Continuous compound interest traffic growth rate *q*_*x*_
1	642.96	
2	720.55	11.39
3	1047.71	37.43
4	1167.35	10.81
5	1487.96	24.27
6	1381.71	-7.41
7	1505.55	8.58
8	1722.03	13.43
9	1840.22	6.64
10	1961.31	6.37
11	2088.26	6.27
12	2253.71	7.62

Source: Research on Subsidy of Highway BOT Project Based on Real Option https://kns.cnki.net/KCMS/detail/detail.aspx?dbname=CMFD201701&filename=1016216268.nh.

where the mean value of *q*_*x*_ is calculated as $\hat{q}$ = 11.43%,

$$\alpha =\frac{1}{X-1}\ln\frac{Q_{X}}{Q_{1}}=\frac{1}{12-1}\ln\frac{Q_{12}}{Q_{1}}=11.4\%$$

$$\sigma =\sqrt{\frac{1}{X-1}\sum_{x=2}^{X}{\left(q_{x}-\bar{q}\right)}^{2}}=\sqrt{\frac{1}{12-1}\sum_{x=2}^{12}{\left(q_{x}-\bar{q}\right)}^{2}}=10.86\%$$

We obtain the mean and variance of the traffic growth rate of continuous compound interest through Monte Carlo simulation with the help of Crystal Ball software. Based on the remaining franchise period, the traffic volume growth rate *q*_*x*_ in year X of each year is sampled 1000 times. Assuming that the project traffic volume in year 1 of the franchise period is 6 million vehicles, the traffic volume *Q*_*X*_ = *Q*_*X*−1_(1+*α*) in year X is calculated at the same time to obtain 1000 simulated traffic volumes and average them. The final average of the traffic volume is the amount of traffic for the remaining franchise years. We repeat the above steps 16 times to obtain the final result (Table 3).

**Table 3:** Summary of simulation data.

Year	Simulation value of traffic growth rate	Simulated traffic volume (10,000 vehicles)
6		600
7	0.1123	667.38
8	0.1159	744.7293
9	0.1119	828.0646
10	0.1155	923.706
11	0.1116	1026.792
12	0.1118	1141.587
13	0.1161	1274.125
14	0.1087	1412.623
15	0.1146	1574.509
16	0.1144	1754.633
17	0.1122	1951.503
18	0.1161	2178.072
19	0.1152	2428.986
20	0.1143	2706.619
21	0.1096	3003.265
22	0.1108	3336.026
23	0.1096	3701.655
24	0.118	4138.45
25	0.109	4589.541

(Source: author’s work).

### 5.2 The simulation results

1) Calculate the static value of the project

The cash inflow is the traffic volume multiplied by the pass price, and according to hypothesis 2, the operating expenses during the operation period are 378.574 million yuan. Therefore, the calculation method of net cash flow is shown in (Table 4).

**Table 4:** Simulation data of this project.

Year	Traffic volume (10,000 units)	Cash inflow (ten thousand yuan)	Cash outflow (ten thousand yuan)	Net cash flow (ten thousand yuan)
1	0	0	150000	-150000
2	0	0	250000	-250000
3	0	0	300000	-300000
4	0	0	150000	-150000
5	0	0	150000	-150000
6	600	43200	37857.44	5342.56
7	667.38	48051.36	37857.44	10193.92
8	744.7293	53620.51	37857.44	15763.07
9	828.0646	59620.65	37857.44	21763.21
10	923.706	66506.83	37857.44	28649.39
11	1026.792	73929	37857.44	36071.56
12	1141.587	82194.26	37857.44	44336.82
13	1274.125	91737.01	37857.44	53879.57
14	1412.623	101708.8	37857.44	63851.38
15	1574.509	113364.7	37857.44	75507.21
16	1754.633	126333.6	37857.44	88476.13
17	1951.503	140508.2	37857.44	102650.8
18	2178.072	156821.2	37857.44	118963.8
19	2428.986	174887	37857.44	137029.6
20	2706.619	194876.6	37857.44	157019.1
21	3003.265	216235.1	37857.44	178377.6
22	3336.026	240193.9	37857.44	202336.5
23	3701.655	266519.2	37857.44	228661.7
24	4138.45	297968.4	37857.44	260111
25	4589.541	330447	37857.44	292589.5

(Source: author’s work).

Therefore, the NPV of the project should be

$$NPV=\sum_{i=0}^{25}\frac{CI_{i}-CO_{i}}{{\left(1+8.6\%\right)}^{i}}=-35.35\mathrm{Billion}$$

The project’s NPV<0. In the traditional NPV calculation method, project investment is no longer meaningful, and the social capital party should quit the project immediately.

2) Calculate the option value of the project
(1) Calculate volatility *σ*

Based on the simulation data of this project, it is assumed that the fixed period of the target project is two years, and the remaining franchise of this project is 20 years. Thus, there are 10 such periods, *n* = 10. The results are calculated according to the calculation formula (8) of historical volatility (Table 5):

**Table 5:** Simulation project value growth rate.

Period *n*	Project value (ten thousand yuan)	*u*_*i*_ (%)
1	8978.442	
2	18504.57	72.3%
3	27110.84	38.2%
4	34909	25.3%
5	42021.41	18.5%
6	48884.27	15.1%
7	55524.01	12.7%
8	61698.58	10.5%
9	67232.42	8.6%
10	73109.72	8.4%

(Source: author’s work).

Therefore, the average value $\hat{u}$ of *u*_*i*_ is 23.3%, and the project volatility *σ* = 20.71%.

(2) Calculate risk-neutral probability *P*

According to the above relevant data, the time interval of the option is Δ*t* = 1. The risk-free rate *r*_*f*_ = 5%, and volatility *σ* = 20.71%. The price-up multiplier $u=e^{\sigma \sqrt{\Delta t}}(u>1)$ = 1.23, and the price-down multiplier *d* = 1/*u*(*d*<1) = 0.81. Thus, risk-neutral probability $P=\frac{e^{r_{f}\Delta t}-d}{u-d}$ = 0.57.

(3) Calculate the option price

The initial value of project *S*_0_ = 12473.65 (million yuan), the strike price *X* = 3929.473 (million yuan), and calculation process of option value are shown in (Table 6).

**Table 6:** Option value calculation process.

Option time	15Δ*t*	14Δ*t*	13Δ*t*	12Δ*t*	11Δ*t*	10Δ*t*	9Δ*t*	8Δ*t*	7Δ*t*	6Δ*t*	5Δ*t*	4Δ*t*	3Δ*t*	2Δ*t*	1Δ*t*
**Option value**	2227.02														
	1459.09	1804.25													
	951.59	1180.31	1461.00												
	616.20	767.97	954.06	1182.36											
	394.55	495.47	619.04	770.47	956.19										
	248.07	315.38	397.63	498.27	621.54	772.65									
	151.27	196.37	251.31	318.38	400.38	500.75	623.73								
	87.29	117.72	154.62	199.50	254.22	321.06	402.82	502.94							
	45.01	65.74	90.71	120.93	157.63	202.31	256.82	323.44	404.98						
	17.07	31.39	48.48	69.01	93.80	123.83	160.33	204.82	259.14	325.57					
	2.71	10.36	21.26	34.98	51.72	72.01	96.59	126.44	162.77	207.08	261.21				
	0.00	1.47	6.22	14.07	24.72	38.15	54.65	74.72	99.12	128.79	164.95	209.09			
	0.00	0.00	0.80	3.70	9.14	17.14	27.70	40.96	57.27	77.16	101.39	130.90	166.91		
	0.00	0.00	0.00	0.43	2.18	5.85	11.69	19.80	30.30	43.44	59.60	79.35	103.43	132.80	
	0.00	0.00	0.00	0.00	0.23	1.28	3.69	7.85	13.94	22.13	32.61	45.65	61.70	81.31	105.26

(Source: author’s work).

The option value of the project is calculated as *C*_*0*_ = 105.26 billion yuan.

(4) The true value of the project

$$V=V_{0}+gV_{1}$$

Calculate the true value of the project, *V* = -35.35+0.35*105.26 = 1.49 billion yuan

Those who choose to escalate their commitment believe that the real value of the project is greater than 0 and that the continuation of investment in the project is necessary. Therefore, in the fifth year of the project, the social capital party chooses to continue to invest in the project, and EOC occurs.

### 5.3 Discussion

Through the use of numerical simulation, the model constructed in the fourth part of this paper for calculation and analysis is used to determine whether a social capital party should opt out or continue to invest at the moment when a decision must be made, and the EOC occurs. Through the collection of data on similar highways in the first 12 years of traffic flow and cash inflows and operation and maintenance costs, we calculated the growth rate and deviation rate of the highway traffic flow. Monte Carlo simulation was performed with Crystal Ball software to simulate the volume of traffic for the remaining franchise period and to calculate the static financial value of the project according to the net present value formula. The calculation results show that NPV<0. According to the traditional project value judgement criteria, the social capital side should withdraw from the project immediately. However, the model established in this paper takes the real option value into consideration and introduces the binary tree model formula to calculate the option value of the remaining franchise period. According to the calculation results, the option value in the remaining concession period is greater than zero, and the actual value of the project is still greater than zero after combining with the NPV value. Therefore, social capital should choose EOC. The numerical simulation results show that combining the static financial value of the project with the option value can more reasonably determine the best time for the social capital to withdraw from the project. This finding also verifies the modification and applicability of the model established in this paper and provides a basis for the reliability of the model.

We combined the NPV and option value of the project as the real value of the PPP project and determined the best time for a social capital party to withdraw from a project. The applicability of the model established in this paper was verified by numerical simulation results that provided a basis for the reliability of the model.

Escalation of commitment always threatens the re-decision-making process of a social capital party in a project, which affects the judgement of the social capital party on the project failure and leads to resource waste. Combined with the action mechanism, influence factors and control measures of commitment escalation, the following suggestions are proposed for the social capital party of a PPP project.

First, reduce the impact of initial responsibility and evaluate the project on a regular basis. Reducing the threat of negative feedback has proven to be an effective way to reduce EOC. At the same time, frequent project review and evaluation can effectively reduce the EOC of failed projects.

Second, it is necessary to clarify the initial objectives of a PPP project and make changes to the senior management personnel when necessary. Due to the long construction and operation cycle of PPP projects, the higher the project completion degree, the more likely some project leaders are to inaccurately judge the project due to personal psychological factors and the more likely they are to choose to escalate their commitment.

Finally, set a minimum goal for the project. One of the important reasons for the EOC is that the social capital party lacks a specific criterion for the success or failure of PPP projects. After setting the minimum goal of the project, when the project is faced with a second decision, the decision maker will have a clear evaluation of the existing value of the project, which prevents the decision maker from falling into a decision-making dilemma due to unclear negative feedback on the project and the occurrence of commitment escalation.

## 6 Conclusion

First, this paper summarizes the research status of commitment escalation and withdrawal of social capital in PPP projects. Then, on the premise of the profit maximization theory, the concept of commitment upgrading is introduced into the PPP project exit timing principle model, and the exit time selection of social capital parties under ideal conditions is compared and analysed through the graphic method. Through the combination of real option theory and commitment escalation theory, a model combining the binary tree option pricing model and NPV is established. Finally, the decision model is further analysed with a specific numerical simulation, and control measures of commitment escalation by a social capital party are proposed. Based on the analysis, the following conclusions can be drawn.

First, the social capital party is one of the three main subjects of a PPP project, and its decision-making behaviour plays an important role in the smooth implementation of the PPP project. However, the decision maker is not a completely idealized individual. Due to various factors, after receiving negative feedback from the project, the decision maker may not make timely exit decisions but may choose to continue to invest resources in the failed project, causing more unnecessary losses.

Second, in the model of the social capital exit timing principle, the impact of EOC theory on PPP projects can be divided into two types: First, it leads investors to more accurately evaluate PPP projects. Second, the influence of prospect theory and marginal effects makes investors’ judgement evaluation of the reinvestment cost of the project biased. Although different theories affect different judgements of investors, compared with the ideal state, the investment period of PPP projects is extended because of the EOC; that is, investors make the decision to continue investment.

Finally, in the decision-making model of exit timing, after comparing the real option with a commitment upgrade, it is found that the commitment upgrade is also a dynamic decision-making method. When a social capital party values the uncertain value of a project, it is likely to choose EOC.

In view of the above conclusions, this paper puts forward the following suggestions for PPP projects:

First, before the government and social capital investors sign a PPP contract, the option value should be included in the scope of project value evaluation. For PPP projects with high option value, the government can improve the participation enthusiasm of social capital by transferring the income distribution right. When the project option value is low, the two sides should establish in advance the project value "red line"; when the project is facing a second decision, decision makers’ existing valuation according to the project and the distance between the "red line" can be more clear, avoiding the problems arising when the understanding of the project negative feedback is not clear and the resulting decision-making dilemma, i.e., EOC.

Second, it is necessary to evaluate the income types of PPP projects scientifically and formulate the withdrawal compensation mechanism in advance. For earnings volatility modelling of a project, in the government and social capital game, both parties should negotiate in advance the clear risk liability on both sides. The exit compensation mechanism should be detailed in the written contract. Parties and social capital should regularly review and evaluate the project to understand the project negative feedback threat and should promise to update this understanding carefully, preventing commitment to escalate failure from causing a greater loss. For projects with stable income, social capital can also strive for feasible subsidies to improve the future value of the project instead of directly withdrawing from PPP projects.

Finally, it is necessary to continue to improve the decision criteria for commitment escalating and provide clear data indicators for decision makers. Due to the long construction and operation cycle of PPP projects, the higher the completion degree of the project, the more likely some project leaders are to make inaccurate judgements of the project due to personal psychological factors, causing them to escalate their commitment. At this time, a scientific and reasonable decision standard is needed to free the decision maker from the constraints of previous failure factors and make a reasonable judgement on the project.
